# Relative Spatial Frequency Processing Drives Hemispheric Asymmetry in Conscious Awareness

**DOI:** 10.3389/fpsyg.2017.00559

**Published:** 2017-04-19

**Authors:** Elise A. Piazza, Michael A. Silver

**Affiliations:** ^1^Princeton Neuroscience Institute, Princeton University, PrincetonNJ, USA; ^2^Helen Wills Neuroscience Institute, University of California, Berkeley, BerkeleyCA, USA; ^3^School of Optometry, University of California, Berkeley, BerkeleyCA, USA; ^4^Vision Science Graduate Group, University of California, Berkeley, BerkeleyCA, USA

**Keywords:** conscious awareness, binocular rivalry, perceptual selection, spatial frequency, hemispheric asymmetry

## Abstract

Visual stimuli with different spatial frequencies (SFs) are processed asymmetrically in the two cerebral hemispheres. Specifically, low SFs are processed relatively more efficiently in the right hemisphere than the left hemisphere, whereas high SFs show the opposite pattern. In this study, we ask whether these differences between the two hemispheres reflect a low-level division that is based on absolute SF values or a flexible comparison of the SFs in the visual environment at any given time. In a recent study, we showed that conscious awareness of SF information (i.e., visual perceptual selection from multiple SFs simultaneously present in the environment) differs between the two hemispheres. Building upon that result, here we employed binocular rivalry to test whether this hemispheric asymmetry is due to absolute or relative SF processing. In each trial, participants viewed a pair of rivalrous orthogonal gratings of different SFs, presented either to the left or right of central fixation, and continuously reported which grating they perceived. We found that the hemispheric asymmetry in perception is significantly influenced by relative processing of the SFs of the simultaneously presented stimuli. For example, when a medium SF grating and a higher SF grating were presented as a rivalry pair, subjects were more likely to report that they initially perceived the medium SF grating when the rivalry pair was presented in the left visual hemifield (right hemisphere), compared to the right hemifield. However, this same medium SF grating, when it was paired in rivalry with a lower SF grating, was more likely to be perceptually selected when it was in the right visual hemifield (left hemisphere). Thus, the visual system’s classification of a given SF as “low” or “high” (and therefore, which hemisphere preferentially processes that SF) depends on the other SFs that are present, demonstrating that relative SF processing contributes to hemispheric differences in visual perceptual selection.

## Introduction

Although we may often be unaware of its impact on our everyday experiences, spatial frequency (SF) information is perceived and processed differently in the left visual field (LVF) and right visual field (RVF), due to differences in perceptual specialization between the two hemispheres (Sergent, 1982; Kitterle et al., 1990; Ivry and Robertson, 1998; Piazza and Silver, 2014). Within a stimulus set, identification and discrimination of low SFs tend to be faster and more accurate for stimuli presented in the LVF, whereas high SFs are more quickly and accurately processed in the RVF. This asymmetry has been observed for both sinusoidal gratings (Christman et al., 1991; Hellige, 1993; Christman, 1997) and SF-filtered natural scenes (Peyrin et al., 2003).

Physiologically, fMRI studies show that areas in the left hemisphere respond preferentially to high SF compared with low SF stimuli, whereas the right hemisphere shows the opposite pattern (Peyrin et al., 2004; Musel et al., 2013). Additionally, EEG responses are larger in the left compared with the right hemisphere for high SF stimuli and larger in the right than the left hemisphere for low SFs (Martínez et al., 2001). Moreover, directing attention to one of two SF components of a grating while preparing to perform either a local- or global-level discrimination of a subsequently presented Navon stimulus differentially modulates the amplitude of alpha-band EEG signals in the two hemispheres (Flevaris et al., 2011). Here, we asked whether the differential filtering of a given SF in the two hemispheres for conscious awareness depends on relative processing of the set of available SFs in the environment at any given time.

Most previous behavioral studies of hemispheric asymmetries in SF processing have relied primarily upon measures of reaction times (RTs) to single stimuli that were briefly flashed in either the LVF or RVF. For example, Kitterle and Selig (1991) found that RTs for SF discrimination of two successively presented sinusoidal gratings were faster for lower SF gratings (1–2 cycles/degree, or cpd) in the LVF and for higher SF gratings (4–12 cpd) in the RVF. One study (Kitterle et al., 1992) used gratings with multiple SF components (a low fundamental frequency and higher harmonics) to compare selective processing of these components in the LVF versus RVF. However, in this study, participants were required to make a single perceptual judgment based on a particular SF component (either “Are the bars wide or narrow?” (low SF) or “Are the bars sharp or fuzzy?” (high SF)), so simultaneous perceptual processing of multiple SF components was never assessed within a given trial.

In a recent study (Piazza and Silver, 2014), we investigated the effects of hemispheric asymmetry on conscious visual representations by measuring perceptual selection from multiple SFs simultaneously present in the environment. Perceptual selection is the process of determining which of multiple possible percepts will be dominant, or consciously perceived, when visual input is consistent with multiple interpretations (e.g., the Necker cube). We used binocular rivalry, a bistable phenomenon in which two incompatible images are presented separately to the two eyes at overlapping retinal locations, resulting in perceptual alternation between the two images, even though the visual stimuli remain constant (Blake and Logothetis, 2002).

In contrast to the previous work described above, our use of binocular rivalry provided a number of advantages for studying hemispheric asymmetries in perceptual selection of SFs. First, binocular rivalry allows a direct measure of the subjective content of dynamic perceptual experience (compared to RT, which merely measures the speed of processing of a particular stimulus). Furthermore, binocular rivalry reflects conscious awareness of one of two competing images at any given moment, without the need to explicitly direct the subject’s attention to a particular SF component, as in previous studies (e.g., Kitterle et al., 1992). In addition, binocular rivalry involves competition between multiple possible perceptual interpretations to resolve ambiguity in the visual inputs, a ubiquitous real-world problem that the visual system constantly faces.

In our previous study, we presented two orthogonal gratings with distinct SFs (1 and 3 cpd) to the same retinal location in the two eyes in either the left or right hemifield. We found that the lower SF grating was perceived more often when it was presented in the left hemifield (right hemisphere), whereas the higher SF showed the opposite pattern (Piazza and Silver, 2014). This result raised the intriguing question of how the visual system assigns SFs to the two hemispheres.

One possibility is that there is an absolute threshold, above which SFs are processed more efficiently by the left hemisphere than the right hemisphere, and below which they are perceived more efficiently by the right hemisphere. This would be consistent with the existence of filters in the visual system that are tuned for particular SFs (De Valois et al., 1982). Alternatively, the hemispheric preference for each SF could be evaluated relative to the other SFs that are simultaneously present in the visual scene. This would suggest the existence of a classification system for SF processing that is updated based on the set of SFs in the visual environment at any given time.

For example, given a natural scene containing predominantly high SFs (e.g., a forest), relative SF processing would result in the lower SFs in the scene (trunks and branches, as opposed to leaves) being processed more efficiently in the left hemifield/right hemisphere. However, those same trunks and branches would represent relatively high SFs and would be processed better in the right hemifield/left hemisphere in a scene with mainly low SFs (e.g., a landscape).

In addition, relative processing of SFs could allow hemispheric specialization for different features to be invariant over a range of distances from the viewed object. For example, the right hemisphere’s preferential involvement in emotional recognition of faces, driven by relatively lower SFs, and the left hemisphere’s preference for face identity information, driven by relatively higher SFs (Vuilleumier et al., 2003), could be maintained for both near and far faces. A hemispheric filtering mechanism based solely on absolute SF would not allow such flexible scaling because both emotion and identity information could be low or high SF, depending on viewing distance.

Using static gratings containing multiple SF components, Christman et al. (1991) found that a SF of 2 cpd was differentially processed in the two hemispheres, depending on whether it was relatively higher or relatively lower than other simultaneously presented SFs. However, this study relied on RTs to presentation of gratings, and not a direct measure of dynamic perceptual experience, to investigate hemispheric asymmetry. Moreover, the task (subjects indicated whether the 2 cpd component was present or absent) may have been influenced by non-perceptual factors (e.g., response criterion bias). Thus, the role of relative SF processing in hemispheric differences in perceptual selection of SFs from the environment is unknown.

In the present study, we investigated the role of relative SF processing in hemispheric asymmetries in visual perception. To do this, we expanded the range of SFs that were competing with each other for conscious awareness in binocular rivalry, such that a given grating was the relatively higher SF in some trials but the relatively lower SF in other trials. We found that the hemispheric preferences for a given SF differ as a function of whether it is the relatively low versus high SF in a rivalry pair. Our results demonstrate a novel influence of relative processing on visual perception, in which flexible selection of SFs in the environment results in preferential and asymmetric processing in the two hemispheres.

## Materials and Methods

### Participants

Fifteen right-handed participants (aged 19–38, 10 women) completed this study. All participants provided written informed consent in accordance with the Declaration of Helsinki, and all experimental protocols were approved by the Committee for the Protection of Human Subjects at the University of California, Berkeley. Each participant completed a 1-h session composed of two blocks. We collected data from 20 participants but excluded five participants’ data sets from analysis. Of the excluded participants, two were missing substantial portions of data due to incorrect response key mapping, one responded incorrectly on more than 25% of the catch trials (see Procedure), and two experienced no perceptual alternations of rivalrous stimuli on a majority (>75%) of trials (perhaps due to strong eye dominance; Dieter et al., 2017). While our study is focused on initial perceptual selection and not on perceptual alternations in binocular rivalry *per se*, perceptual alternations are a defining characteristic of binocular rivalry, and it was unclear that these two subjects experienced binocular rivalry in a typical manner.

### Visual Stimuli

**FIGURE 1:**
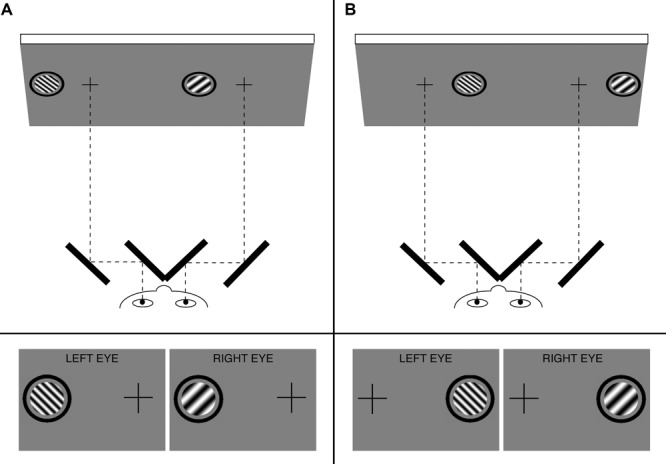
**Top: Schematic of an example visual display and mirror stereoscope in (A)** the left visual hemifield condition and **(B)** the right visual hemifield condition. Bottom: Example images (from Pair 2; see **Figure 2A**) presented dichoptically to the two eyes. Participants maintained fixation on a binocularly presented cross while continuously reporting their perception of rivalrous gratings that were presented to either the left **(A)** or the right **(B)** of the fixation cross. Permission to reproduce figure (slightly modified from Piazza and Silver, 2014) was granted from MIT Press.

Binocular rivalry stimuli were generated on a Macintosh PowerPC using MATLAB and Psychophysics Toolbox (Kleiner et al., 2007) and were displayed on a gamma-corrected NEC MultiSync FE992 CRT monitor with a refresh rate of 60 Hz at a viewing distance of 100 cm. Participants viewed all stimuli through a mirror stereoscope with their heads stabilized by a chin rest. Stimuli were monochromatic circular patches of sine wave grating 1.8° in diameter that were surrounded by a black annulus with a diameter of 2.6° and a thickness of 0.2° (**Figure 1**). Binocular presentation of this annulus allowed it to serve as a vergence cue to stabilize eye position. The stimuli were presented on the horizontal meridian, centered at 3.5° eccentricity either to the left or right of a black central fixation cross. Because the fixation crosses were in the same location on the screen in both hemifield conditions (**Figure 1**, top), participants’ eye position, relative to the head, was the same in both conditions. All gratings were presented at 100% contrast and had the same mean luminance as the neutral gray background (59 cd/m^2^).

**FIGURE 2:**
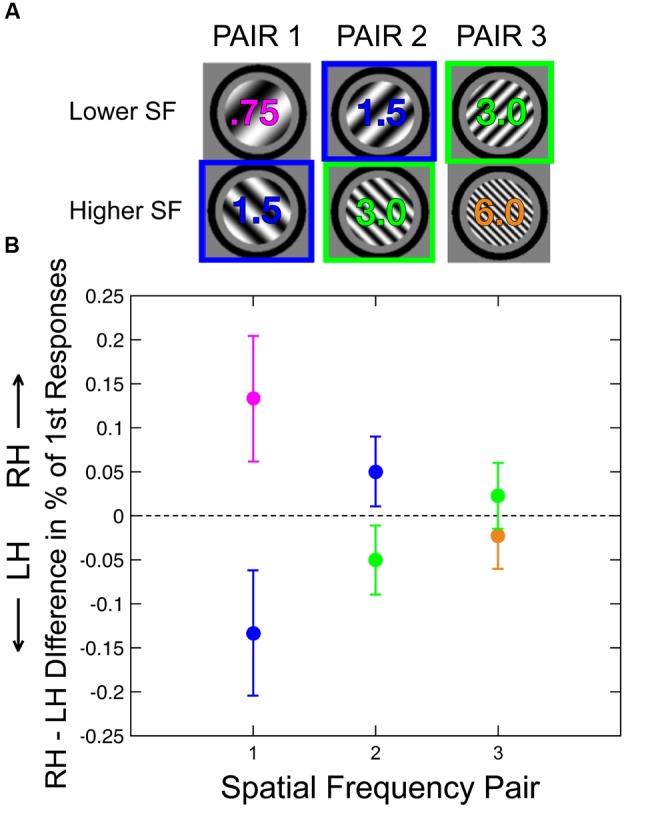
**(A)** The three rivalry pairs used in this study, one of which was presented in each trial. Each color indicates a different absolute spatial frequency. **(B)** Difference in perceptual selection between the two hemispheres for each rivalry pair. Positive values correspond to a higher proportion of initial percepts in the RH (right hemisphere, or left visual field) condition, and negative values correspond to a higher proportion of initial percepts in the LH (left hemisphere, or right visual field). The hemispheric preference for a given grating (e.g., the 1.5 cpd grating) changed as a function of the SF of the rivaling grating (e.g., the blue data points for the 1.5 cpd grating are on opposite sides of the dotted line in Pair 1 vs. Pair 2), indicating that relative SF processing contributes to hemispheric asymmetry in perceptual selection. *N* = 15. Error bars are within-subject SEM across participants.

In all trials except for the catch trials (see Procedure), the two sinusoidal gratings had different SFs, corresponding to the values depicted in one of the columns of **Figure 2A**. Specifically, each pair of SFs was one of the following: 0.75/1.5 cpd, 1.5/3 cpd, or 3/6 cpd. We refer to the relatively lower SF in each pair as the LSF and the relatively higher SF as HSF. The two gratings in a pair were orthogonal, with ±45° orientations relative to vertical. The SF and orientation of the grating presented to each eye were fully counterbalanced and randomly selected across trials.

### Procedure

Before starting the experiment, each participant adjusted the stereoscope by rotating its mirrors until the two eyes’ images (**Figure 1**, bottom, with the orthogonal gratings replaced by identical figures in both eyes for this adjustment phase) were fused, allowing the participant to perceive a single cross and annulus with binocular viewing. All participants completed five practice trials in each hemifield condition before starting the experiment to ensure that they were using the correct response keys and that the stereoscope was properly aligned.

In each trial, the static gratings, fixation cross, and annuli (**Figure 1**) were presented continuously for 10 s, with a 1500-ms blank interval (consisting of only the fixation cross and annuli) between trials. A brief (250 ms) pure tone auditory cue was presented immediately before the onset of the grating stimuli to signal the beginning of each trial. Throughout each trial, participants used one of two keys to indicate their percept: either a grating tilted to the left or a grating tilted to the right. We asked participants to report tilt, a feature orthogonal to the dimension of interest (SF), to reduce the likelihood of response bias. Participants were instructed to continuously press a key with their right hand for as long as the corresponding percept was dominant and to not press any key for ambiguous percepts. The experiment was separated into two blocked conditions: left hemifield and right hemifield, the order of which was counterbalanced across participants. Each participant completed 96 trials and 8 catch trials per hemifield condition.

Catch trials, in which the gratings presented to the two eyes were identical in every way (e.g., both 1.5 cpd and ±45°), were randomly interleaved with the normal rivalry trials throughout the experiment to determine whether participants were accurately reporting their percept and using the correct response key mapping. In these catch trials, gratings were presented statically for the entire duration of each trial. As in the experimental trials, participants were asked to continuously report what they saw throughout the trial, and responses were measured over the entire trial duration. Participants who responded incorrectly (i.e., made at least one key press corresponding to the tilt that was orthogonal to that of the presented gratings) in more than 25% of the catch trials were excluded.

## Results

Immediately after presentation of a pair of stimuli in binocular rivalry, participants often experience an ambiguous percept (consisting of a patchwork or mixture of the two images), followed by a perceptual alternation between two distinct images. We defined the initial response on each trial as the first key press by the subject, as this indicates the subject’s first percept that clearly corresponded to one of the two orthogonal gratings. On average, this initial response occurred 1.6 s after the start of the trial. Only 3% of the total trials across participants contained any response in the first 300 ms, suggesting that automatic responses occurred very infrequently, if at all.

For each subject, we measured the proportion of initial responses corresponding to either the LSF (relatively lower) or HSF (relatively higher) grating for each of the three SF pairs (**Figure 2A**). Initial responses were recorded for both visual hemifields, so every subject contributed three pairs of scores for the LVF and three pairs for the RVF. To evaluate hemispheric differences in SF processing, we subtracted the proportion of initial responses in the RVF (left hemisphere, or LH) from that in the LVF (right hemisphere, or RH) for each SF (**Figure 2B**). Because the two plotted difference values for a given SF pair are always identical in magnitude but opposite in sign (due to the complementary nature of responses, which always corresponded to either the LSF or HSF stimulus), we only analyzed the LSF responses.

A one-way ANOVA indicated that there was no significant main effect of SF pair on the magnitude of the hemispheric difference score [*F*(2,14) = 1.13, *p* = 0.34]. The assumption of equality of variances was met for this ANOVA and for all other ANOVAs reported in this paper (Levene’s test, *p* > 0.10 in all cases). We therefore averaged the three hemispheric difference scores for each subject (**Figure 2B**) for the LSF stimuli (top row of gratings in **Figure 2A**) to estimate the overall hemispheric bias across all three stimulus pairs. A positive score corresponds to a RH bias for the relatively lower SF in a pair (and a LH bias for the HSF), a negative score corresponds to a LH bias for the LSF, and a score of 0 corresponds to no difference between the hemispheres in processing the two SFs in a pair. We found that this overall score was significantly greater than zero across subjects (two-sided one-sample Wilcoxon signed-rank test; *p* < 0.02), indicating that hemispheric differences in initial perceptual selection of SFs depend on relative frequency processing.

For each of the two SFs that appeared in two different grating pairs (1.5 and 3 cpd, shown in blue and green, respectively, in **Figure 2A**), we also compared the hemispheric difference score (**Figure 2B**) for the two SFs with which it had been paired (for 1.5 cpd, paired with either 0.75 or 3 cpd; for 3 cpd, paired with either 1.5 or 6 cpd). Specifically, for each SF, we compared the within-subject hemispheric difference score between trials in which it was the relatively lower SF in a pair and the trials in which the *same grating* was the relatively higher SF in a pair. This procedure enabled us to directly quantify the contribution of relative SF processing to hemispheric asymmetry in perceptual selection. We found a significant main effect of whether a given SF was the relatively higher or lower member of a rivalry pair on hemispheric difference scores across the two analyzed SFs (1.5 and 3 cpd) [two-way ANOVA, *F*(1,14) = 5.59, *p* < 0.05]. In addition, there was not a significant interaction between this factor and absolute SF (1.5 vs. 3 cpd) [*F*(1,14) = 0.65, *p* = 0.80], indicating that the magnitude of the relative SF processing effect did not differ significantly between the two analyzed SFs.

**FIGURE 3:**
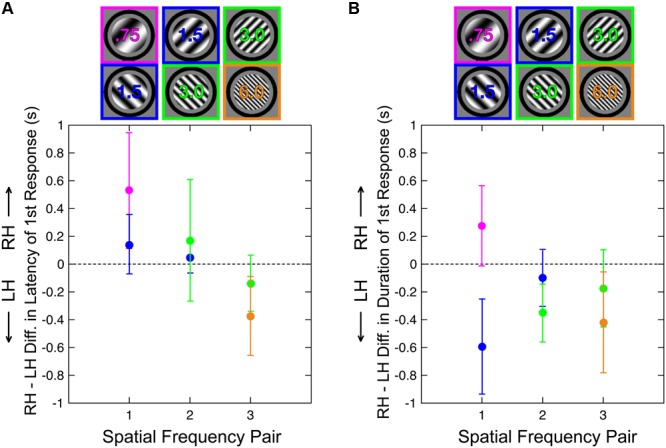
**Hemispheric difference scores for the (A)** latency and **(B)** duration of initial percepts. Each color represents a different absolute SF. Positive values correspond to **(A)** longer initial latencies (slower responses) and **(B)** longer initial percepts in the RH, and negative values correspond to longer latencies (and percepts) in the LH. *N* = 15. Error bars are within-subject SEM across participants.

Finally, we also analyzed the latency and duration of the initial response in each trial (**Figure 3**). For each measure, we again computed a hemispheric difference score (i.e., RH–LH latency, RH–LH duration). We analyzed LSF and HSF separately here because these measures, unlike proportion of initial responses, are not complementary. One-way ANOVAs indicated that there were no significant main effects of SF pair on hemispheric differences in latency of initial responses for either LSF [*F*(2,14) = 1.56, *p* = 0.23] or HSF [*F*(2,14) = 0.64, *p* = 0.54] gratings or on the hemispheric differences in duration of initial responses for either LSF [*F*(2,14) = 1.35, *p* = 0.28] or HSF [*F*(2,14) = 0.19, *p* = 0.82] gratings. We therefore averaged the three hemispheric difference values for each subject for the LSF stimuli and for the HSF stimuli to estimate the overall hemispheric biases across all three grating pairs.

This average hemispheric difference score (RH–LH) was not significantly different from 0 for latencies of LSF (Wilcoxon signed-rank test; *p* = 0.39) or HSF (*p* = 0.93) perceptual reports (**Figure 3A**). The same average hemispheric difference score (again, RH–LH) was not significantly different from 0 for duration of LSF initial responses (*p* = 0.93), but durations of HSF responses were significantly greater in the LH than the RH (*p* < 0.02; **Figure 3B**), a pattern consistent with results reported in Piazza and Silver (2014).

The trial duration we used here (only 10 s, compared to 30 s in our previous paper, Piazza and Silver, 2014) was designed to investigate the properties of initial responses. Because binocular rivalry typically involves a perceptual alternation, the second response strongly depends on the first response. With sufficiently long trial duration, the influence of the first percept on subsequent responses diminishes, but in our study, the relatively short trials preclude meaningful analysis of responses after the initial response.

## Discussion

We have found that differences in perception of basic visual information between the two cerebral hemispheres are influenced by the set of SFs that is present in the environment at any given time. Our findings extend previous reports of hemispheric asymmetries in processing of SFs, as measured with RT (Christman et al., 1991), fMRI (Peyrin et al., 2004), EEG (Martínez et al., 2001), and perceptual selection (Piazza and Silver, 2014), by showing that the hemispheric asymmetry in perceptual selection of a given SF depends on the other SFs that are simultaneously present.

To the best of our knowledge, the origin and adaptive value of hemispheric asymmetries in SF processing remain somewhat mysterious. There has been some speculation that these asymmetries have developed in conjunction with literacy, in particular because text contains relatively high SFs, language-related areas (e.g., visual word form area) are generally lateralized to the left hemisphere, and literate and non-literate individuals differ in the degree of lateralization (Lecours et al., 1988). Future work investigating perceptual selection of broadband naturalistic stimuli may help to elucidate the evolutionary origin of the role of relative SF processing in hemispheric asymmetries.

Our findings are generally related to previous work on contextual modulation in that perception of a stimulus is influenced by other stimuli present in the visual scene. However, unlike contextual effects that arise from interactions between stimuli at different spatial locations (reviewed in Albright and Stoner, 2002; Wagemans et al., 2012), the novel modulation of perceptual selection that we report here is based on a comparison between two SFs, presented in the same retinal location but to different eyes, that compete for visual awareness. This dependence of perceptual experience on a comparison, or integration, of information between two distinct images that are presented simultaneously to different eyes is similar to established effects of interocular Gestalt grouping on binocular rivalry (Díaz-Caneja, 1928; Kovács et al., 1996; see Bressler et al., 2013, for a review).

Because the hemispheric preference for a given SF in our study depends on the SF with which it is paired, our results cannot be explained solely by bottom-up filtering based on a simple absolute SF threshold applied to each grating and thus likely involve some higher-order integration of the rivaling gratings. However, there may be additional mechanisms based on absolute SF that also influence perceptual selection. For example, we have found in the present study that in general, responses to LSFs are more prevalent and faster than responses to HSFs (data not shown), replicating our previous finding that low SFs tend to dominate high SFs (Piazza and Silver, 2014). Moreover, low SF stimuli are more strongly suppressed than high SF stimuli in continuous flash suppression (Yang and Blake, 2012). It should be noted that even these effects might be influenced by relative processing that is based on continuous recalibration in response to the current set of SFs in the visual scene.

As we did not measure eye position, it is possible that subjects made some eye movements toward the peripheral gratings. However, our overall pattern of results is very unlikely to be explained by eye movements. First, we find that LSF initial percepts were generally more prevalent than HSF percepts (Wilcoxon signed-rank test, comparing likelihood of initially responding to 0.75 or 1.5 cpd versus 3 or 6 cpd, *p* < 0.001), and they also have shorter latencies (same comparison, *p* < 0.05). These results are consistent with measurements of initial perception in binocular rivalry that were reported in Piazza and Silver (2014), whereas previous work (Baddeley and Tatler, 2006) demonstrated that eye movements tend to be drawn toward HSF content.

Second, previously reported attentional biases toward the LVF (Siman-Tov et al., 2007) could not explain how each SF’s hemifield bias changes across different SF pairs, as our primary hemispheric difference measure is symmetric across the hemifields for a given SF pair (**Figure 2B**). Third, the combination of attentional biases for the LVF and HSFs presumably result in an increased probability of higher SF initial percepts for LVF stimuli, which is exactly the opposite of the pattern of results that we observed. Finally, if participants were frequently making eye movements to the peripheral gratings, thereby placing them at foveal locations, this would be likely to reduce the measured differences between the LVF and RVF in SF processing.

Our results suggest that the visual system classifies SFs that are present in the current visual environment and preferentially routes them for processing into the left or right hemisphere. Future work might explore the neural mechanisms of this routing (e.g., which brain areas have responses that reflect relative SF processing, the role of top-down feedback from within or beyond the visual system) and how temporal context (i.e., recent experience with scenes containing low vs. high SFs) may influence hemispheric preferences for a given SF. Another important future direction for research is to reconcile the well-established symmetries in visual field representations in the two hemispheres (i.e., the many cortical areas that contain contralateral representations of the visual field; Silver and Kastner, 2009) with the hemispheric asymmetry in relative processing of SFs that we report here.

## Author Contributions

EP designed the study, collected and analyzed the data, and wrote the paper. MS designed the study and wrote the paper.

## Conflict of Interest Statement

The authors declare that the research was conducted in the absence of any commercial or financial relationships that could be construed as a potential conflict of interest.
